# Invasive Nontuberculous Mycobacterial Infections among Cardiothoracic Surgical Patients Exposed to Heater–Cooler Devices^1^

**DOI:** 10.3201/eid2305.161899

**Published:** 2017-05

**Authors:** Meghan M. Lyman, Cheri Grigg, Cara Bicking Kinsey, M. Shannon Keckler, Heather Moulton-Meissner, Emily Cooper, Minn M. Soe, Judith Noble-Wang, Allison Longenberger, Shane R. Walker, Jeffrey R. Miller, Joseph F. Perz, Kiran M. Perkins

**Affiliations:** Centers for Disease Control and Prevention, Atlanta, Georgia, USA (M.M. Lyman, C. Grigg, C. Bicking Kinsey, M.S. Keckler, H. Moulton-Meissner, M.M. Soe, J. Noble-Wang, J.R. Miller, J.F. Perz, K.M. Perkins);; Pennsylvania Department of Health, Harrisburg, Pennsylvania, USA (C. Bicking Kinsey, A. Longenberger, J.R. Miller);; WellSpan York Hospital, York, Pennsylvania, USA (E. Cooper, S.R. Walker)

**Keywords:** NTM, nontuberculous mycobacteria, cardiac surgery, cardiopulmonary bypass, heater–cooler device, bacteria, tuberculosis and other mycobacteria

## Abstract

Invasive nontuberculous mycobacteria (NTM) infections may result from a previously unrecognized source of transmission, heater–cooler devices (HCDs) used during cardiac surgery. In July 2015, the Pennsylvania Department of Health notified the Centers for Disease Control and Prevention (CDC) about a cluster of NTM infections among cardiothoracic surgical patients at 1 hospital. We conducted a case–control study to identify exposures causing infection, examining 11 case-patients and 48 control-patients. Eight (73%) case-patients had a clinical specimen identified as *Mycobacterium avium* complex (MAC). HCD exposure was associated with increased odds of invasive NTM infection; laboratory testing identified patient isolates and HCD samples as closely related strains of *M. chimaera*, a MAC species. This investigation confirmed a large US outbreak of invasive MAC infections in a previously unaffected patient population and suggested transmission occurred by aerosolization from HCDs. Recommendations have been issued for enhanced surveillance to identify potential infections associated with HCDs and measures to mitigate transmission risk.

Nontuberculous mycobacteria (NTM) typically cause infection in patients who are immunocompromised or have chronic lung disease (*1*–*5*) but have also caused healthcare-associated infections related to water sources such as showers and ice machines (*6*–*8*). Outbreaks of NTM infections have occurred among patients undergoing cardiac surgery; these typically involve surgical site infections or infections associated with contaminated products, such as prosthetic implants and cardioplegia solutions (*6*,*7*,*9*). Pulmonary infections are the most common disease manifestation of NTM, but 10% of NTM infections are extrapulmonary (*2*). Disseminated infections are uncommon among immunocompetent patients (*10*–*15*) but are often serious and require treatment with a long, complicated regimen of antibiotic drugs (*2*).

During spring 2015, investigators in Switzerland reported an outbreak of invasive infections with *Mycobacterium chimaera*, a distinct species within the NTM category *M. avium* complex (MAC), associated with contaminated heater–cooler devices (HCDs) used during cardiopulmonary bypass for cardiac surgery (*16*). HCDs regulate the temperature of patient blood, cardioplegia solution, and warming/cooling blankets through a water circuit not intended to have contact with patients or their blood. Given this outbreak and similar outbreaks reported in other countries in Europe, European public health authorities have issued a warning regarding the risk for *M.* *chimaera* infections associated with HCDs (*17*).

In July 2015, a cluster of invasive NTM infections was identified among patients who underwent cardiothoracic surgery at Wellspan York Hospital in York, Pennsylvania, USA. The Pennsylvania Department of Health (PADOH) and the Centers for Disease Control and Prevention (CDC) conducted a field investigation to identify the extent of infections and determine associated risk factors and exposures to prevent further infections.

## Methods

### Setting

Wellspan York Hospital is a 585-bed community teaching hospital at which ≈650 cardiac surgeries are performed annually. Of these, ≈400 require cardiopulmonary bypass, which involves use of a HCD. Three operating rooms are used for cardiothoracic surgery.

### Initial Case Finding

We searched a database of microbiology results to identify all NTM-positive blood, sputum, pleural fluid, and tissue specimens at this hospital during the previous 5.5 years (January 1, 2010, to July 1, 2015). We cross-referenced patients with an NTM-positive specimen with the hospital’s surgical database to determine whether they underwent surgical procedures during an exposure period 30 days to 3.5 years preceding the NTM-positive specimen collection date. Surgical procedures occurring <30 days before an NTM-positive specimen was collected were excluded because of the likelihood that they were either diagnostic or therapeutic procedures for a suspected NTM infection (and therefore not responsible for NTM transmission). Surgical procedures occurring >3.5 years before an NTM-positive specimen was collected were excluded because available published reports suggested that most NTM infections were diagnosed within 3.5 years after cardiac surgery (*16*). To explore whether NTM infection rates differed by surgery category, we calculated the rate of NTM-positive patients (per 10,000 operations performed) for the 3 most common surgical categories (cardiothoracic, general surgery, or orthopedic) and compared these rates using the Fisher exact test.

### Case–Control Study

We found that NTM-positive specimens occurred at a higher rate among cardiothoracic surgical patients than among patients in other major surgical categories. Given this finding and recent reports suggesting that HCDs are a potential risk factor for NTM infection, we conducted a case–control study to identify risk factors associated with invasive extrapulmonary NTM infections among patients who underwent cardiothoracic surgery at Wellspan York Hospital.

### Case Definition

Inclusion criteria for case-patients were an extrapulmonary NTM-positive specimen collected during 2010–2015 and a cardiothoracic surgery during 2009–2014 occurring during the exposure period (30 days–3.5 years before collection of the NTM-positive specimen). We excluded patients with NTM-positive specimens collected before 2010 because acid-fast bacillus tissue cultures before 2010 were not included in the microbiology database; patients with a history of MAC infection (or a MAC-positive specimen) before cardiothoracic surgery, which suggests that their infection could not be temporally attributed to their cardiac surgery; patients whose cardiothoracic surgeries occurred before 2009, because surgical documentation in the electronic medical record at that time was less standardized and reliable; and patients with only NTM-positive pulmonary specimens, because patients with pulmonary infections have been shown to differ epidemiologically from patients with other types of NTM infections (*11*,*18*). All patients with NTM-positive specimens from an extrapulmonary sterile body site were included.

### Control Selection

We selected 48 unmatched controls at random from a list of all patients who underwent cardiothoracic surgery at this hospital during 2009–2014 and who had no history of MAC infection. Because controls did not have an NTM-positive specimen date to determine the surgical exposure period (30 days–3.5 years before the NTM-positive specimen collection date), we assigned an index date based on the median incubation period of all patients with NTM infection (397 days from cardiothoracic surgery to NTM-positive specimen) and used this date to determine a comparable exposure period.

### Data Collection

We abstracted patient demographic characteristics, medical history or risk factors, outcomes, and NTM specimen information (for case-patients only) from patients’ electronic medical records. We also collected perioperative and hospital exposures for every surgery that patients underwent during the exposure period. For the 1 control-patient who had 2 cardiothoracic surgical procedures that required a cardiopulmonary bypass machine to be operational in the room, we summed time of surgery and time connected to the bypass machine to reflect cumulative exposure for both operations.

### Infection Control, Environmental, and Laboratory Assessment

We conducted interviews with healthcare personnel and directly observed operating room practices during cardiac surgery. We reviewed the facility’s perioperative protocols and the HCD manufacturer’s instructions for use. Before the field team’s arrival, all 3 HCDs used for cardiothoracic surgery at Wellspan York Hospital had been removed from service and replaced with new ones. During the field investigation, we collected water samples from the decommissioned HCDs, the new HCDs introduced during the investigation, the nearest scrub sink, and 2 ice machines that supplied nonsterile ice to the operating rooms. We collected swab samples from the internal water reservoirs of the 3 HCDs. We disassembled 1 HCD to permit a more thorough inspection. Another HCD was operated in an empty cardiothoracic operating room during a simulation in which we collected water samples from the HCD and air samples from various locations within the operating room (18 inches from the HCD exhaust vent and next to the operating room exhaust vents located in the room corners) in 200-L and 500-L volumes using an impaction air sampler (SAS 90; Bioscience International, Rockville, MD, USA) before starting the HCD and then intermittently over 5 hours after starting the HCD. 

Three case-patient isolates (2 from blood and 1 from bone marrow) were available for further characterization. Patient isolates and environmental samples were sent to CDC for testing, including culture isolation, acid-fast bacillus staining, identification by matrix-assisted laser desorption/ionization time-of-flight mass spectrometry and 16S and rpoB sequencing, as well as molecular typing by pulsed-field gel electrophoresis (PFGE) and whole-genome sequencing. 

### Statistical Analyses

We compared patient demographic and clinical characteristics between case-patients and control-patients using the Fisher exact test for categorical variables and 2-sample *t* test and Wilcoxon 2-sample test for continuous variables. To assess the association between case status and surgical exposures, we initially conducted univariable logistic regression with Firth’s penalized maximum likelihood method that accounts for small sample size, issues of data separability, and bias of the parameter estimates, and obtained crude odds ratios (ORs), 95% CIs, and p values. Several surgical exposure variables associated with increased odds of case status were further tested for association with exposure to cardiopulmonary bypass with HCD using the Fisher exact test. Because undergoing a surgery requiring cardiopulmonary bypass with HCD was associated with major cardiothoracic surgery (p<0.0001), presence of a central line (p<0.0001), and implantation of an artificial valve or graft (p<0.0001), we did not include these variables in the multivariable regression analysis that examines the association between case status and length of HCD exposure.

To examine the association between case status and length of HCD exposure, we used 2 different, but related, exposure length variables: surgical time with HCD (time a patient is in the operating room while an HCD is operated) and time on cardiopulmonary bypass (time when a patient’s blood is routed through the bypass machine). We conducted multivariable logistic regression with Firth’s penalized maximum likelihood method to evaluate such relationships and examined collinearity between factors considered in the multivariable models. Because of the notable correlation detected between surgical time with HCD and time on bypass (Pearson ρ = 0.9, variance decomposition proportion for surgical time with HCD = 0.92, and variance decomposition proportion for pump time = 0.88 at a given condition index of 10), we analyzed these 2 exposure-length variables in separate models. For each exposure-length variable, we began with a full saturated model that also included several potential patient factors, all of which were removed by using a backward elimination method owing to nonsignificance at an α of 0.05, except for immunocompromised status, which was retained because of the biological plausibility of this medical condition affecting the risk for invasive NTM infections. We used SAS statistical software version 9.3 (SAS Institute, Inc., Cary, NC, USA) for all analyses.

## Results

### Initial Case Finding

Among 144 patients with an NTM-positive specimen collected during January 1, 2010, to July 1, 2015, 48 (33%) underwent >1 surgery during the exposure period. The rate of NTM-positive specimens was noticeably higher among cardiothoracic surgical patients (20 patients/10,000 surgeries) than the rates among patients in other common surgical categories, including general surgery (8 patients/10,000 surgeries; p = 0.04) and orthopedic surgery (5 patients/10,000 surgeries; p = 0.004). Approximately 2,276 surgical procedures with HCDs were performed during this period.

**Figure 1:**
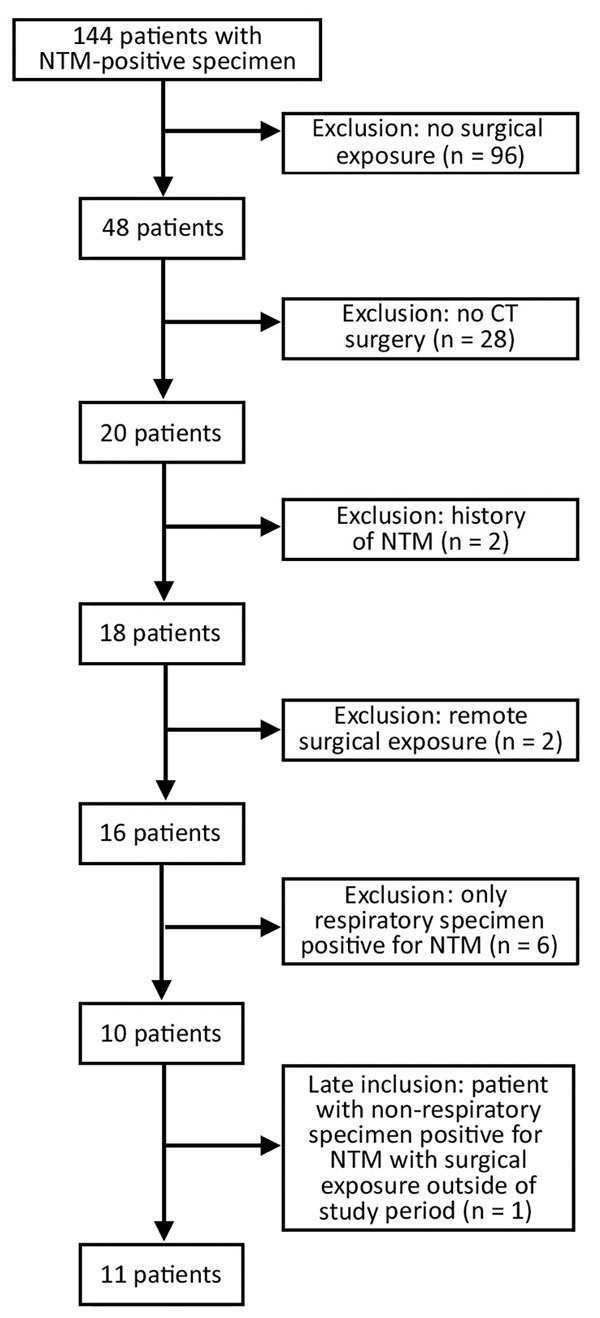
Inclusion criteria flowchart for case–control study of patients with NTM-positive specimens in investigation of invasive extrapulmonary NTM infections among patients who underwent cardiothoracic surgery, York, Pennsylvania, USA, 2015. CT, cardiothoracic; NTM, nontuberculous mycobacteria.

Of the 20 NTM-positive patients who had >1 prior cardiothoracic surgery, we excluded 10 patients based on the case definition (Figure 1), which left 10 patients with invasive extrapulmonary infections, all of whom demonstrated clinical signs of infection at the time of specimen collection. One of the patients who was excluded because of a previous cardiothoracic surgery before 2009, when surgical documentation in the electronic medical record was less standardized and reliable, was later included in the analysis to increase the number of cases, given the low sample size. Demographic and clinical details of these 11 case-patients are shown in Table 1. Most case-patients (8, 73%) had specimens positive for MAC. Five (45%) case-patients were considered to have a thoracic infection, with only specimens from sterile thoracic sites testing positive for NTM. The remaining 6 (55%) case-patients had extrathoracic infections with NTM-positive specimens obtained from sterile body sites outside the thoracic cavity, which likely represented disseminated infections. A large proportion (63%) of patients died, although the cause of death was not necessarily attributable to NTM infection. Of the 11 patients who met the case criteria, 0–3 cases occurred each year during 2010–2015. The median infection latency (length of time between patients’ most invasive cardiothoracic surgery and NTM-positive specimen collection date) was 1.2 years (range 0.1–2.3 years).

### Case–Control Study

Case-patients and control-patients did not differ noticeably on demographics (age, gender, or race) or predisposing medical conditions (chronic lung disease, diabetes, or immunocompromised state) (Table 2). However, sarcoidosis was more likely to have been diagnosed in case-patients than in control-patients (27% versus 0%; p = 0.005).

**Table 2:** Descriptive characteristics of case-patients with invasive extrapulmonary NTM infections and control-patients among patients who underwent cardiothoracic surgery, York, Pennsylvania, USA, 2015*

Characteristic	Case-patients, n = 11	Control-patients, n = 48	p value†
Demographics			
Age, y, mean (range)	69 (47–84)	64 (20–84)	0.33
Male sex	9 (82)	27 (56)	0.17
White race	11 (100)	45 (94)	1.00
Predisposing medical condition			
Chronic lung disease	4 (36)	8 (17)	0.21
COPD	1 (9)	8 (17)	1.00
Sarcoidosis	3 (27)	0	0.005
Diabetes	1 (9)	15 (31)	0.26
Immunocompromised	3 (27)	10 (21)	0.69
HIV	0	0	NA
Transplant	0	0	NA
Chemotherapy	2 (18)	9 (19)	1.00
Steroid treatment	0	2 (4)	1.00
Hematologic malignancy‡	2 (18)	1 (2)	0.09

*Values are no. (%) patients except as indicated. COPD, chronic obstructive pulmonary disease; NA, not applicable; NTM, nontuberculous mycobacteria. †Fisher exact test for categorical variables or two 2-sample *t* tests with pooled variance. ‡Among cases, hematologic malignancy included chronic lymphocytic leukemia and myelodysplastic syndrome. Among controls, hematologic malignancy included Hodgkin’s lymphoma.

Overall, the number of surgical exposures, and specifically cardiothoracic surgical exposures, was similar among case-patients and control-patients (Table 3). Case-patients had 1–3 cardiothoracic surgeries during the exposure period, but none had >1 cardiothoracic surgery requiring cardiopulmonary bypass. Case-patients had greater odds of major cardiothoracic surgery compared with control-patients; all case-patients had undergone either major cardiac surgery or major thoracic surgery. Having major cardiac surgery was associated with increased odds of invasive NTM infection (OR 4.9, 95% CI 1.2–27.5; p = 0.04). Specifically, having aortic surgery increased the odds of being a case-patient 82-fold (95% CI 3.2–>999.9; p = 0.008). Whereas 45% of case-patients had aortic surgery, none of the control-patients had undergone this procedure, likely reflecting its rarity (n = 154, 1.6% of cardiothoracic surgeries). Nine (82%) case-patients had cardiothoracic surgery requiring cardiopulmonary bypass, and most (8, 89%) also had a specimen positive for MAC (Table 1). Having a central line placed during admission (OR 5.5, 95% CI 1.1–53.5; p = 0.07) and having an artificial valve or graft implanted during cardiothoracic surgery (OR 10.1, 95% CI 2.6–48.1; p = 0.002) were both associated with higher odds of invasive NTM infection. Use of cardiopulmonary bypass, which results in exposure to an operating HCD, was also associated with significantly higher odds of invasive NTM infection (OR 5.3, 95% CI 1.3–29.2; p = 0.03). The use of cardiopulmonary bypass with an HCD was correlated with undergoing major cardiothoracic surgery (p<0.0001), placement of a central line (p<0.0001), and implantation of an artificial vale or graft (p<0.0001); these factors were not independently associated with case status. The mean surgical time when an HCD was required and mean time on bypass machine were both significantly longer for case-patients than for controls (surgical time, p = 0.003; time on bypass, p = 0.002).

**Table 3:** Surgical exposures of case-patients with invasive extrapulmonary NTM infections and control-patients among patients who underwent cardiothoracic surgery, York, Pennsylvania, USA, 2015*

Exposures	Case-patients, n = 11	Control-patients, n = 48	Odds ratio (95% CI)	p value†
No. surgeries per patient				
All surgeries, median (IQR)	1 (1–4)	2 (1–3)		0.90
Cardiothoracic surgeries, median (IQR)	1 (1–3)	1 (1–2)		0.86
Type of procedure‡				
Major cardiothoracic procedure	11 (100)	31 (65)	12.8 (1.5–>999.9)	0.09
Major cardiac procedure	9 (82)	21 (44)	4.9 (1.2–27.5)	0.04
Coronary artery bypass grafting surgery	6 (55)	17 (35)	2.1 (0.6–7.9)	0.25
Cardiac valve surgery	4 (436)	8 (67)	2.9 (0.7–11.4)	0.15
Aortic surgery	5 (45)	0 (0)	82.1 (7.9–>999.9)	0.008
Pericardial window	0 (0)	2 (4)	0.8 (0.01–10.9)	0.91
Major thoracic procedure	2 (18)	11 (23)	0.9 (0.1–3.6)	0.85
Perioperative exposures§				
Central line¶	10 (91)	27 (56)	5.5 (1.1–53.5)	0.07
Chest tube	10 (91)	31 (65)	3.9 (0.8–38.2)	0.15
Shower during hospitalization	7 (64)	20 (42)	2.3 (0.7–9.2)	0.21
Artificial valve or graft	8 (73)	9 (19)	10.1 (2.6–48.1)	0.002
Topical medication	10 (91)	37 (77)	2.1 (0.4–21.4)	0.43
Topical antibiotic	10 (91)	31 (65)	3.9 (0.8–38.2)	0.15
Topical anticoagulant	5 (45)	15 (31)	1.8 (0.5–6.7)	0.37
Use of cardiopulmonary bypass with HCD exposure	9 (82)	20 (42)	5.3 (1.3–29.9)	0.03
Length of HCD-related exposures#				
Surgical time with HCD, min, median (IQR)	328 (164–360)	0 (0–222)		0.003
Time on bypass, min, median (IQR)	147 (46–175)	0 (0–74)		0.002

*Any surgery that occurred 30 d to 3.5 y before first NTM-positive specimen for cases or index date for controls. Values are no. (%) patients except as indicated. NTM, nontuberculous mycobacteria. †Fisher exact test for categorical variables or Wilcoxon 2-sample test for continuous variables. ‡Major cardiac surgery: coronary artery bypass grafting, valve replacement, aortic graft, pericardial window; major thoracic surgery: lung removal, thoracotomy, esophagectomy.  §Exposure was considered present if any of the surgeries (>1) had the exposure. ¶Present at any time during hospital stay. Did not include chronic indwelling lines, such as PICC lines or dialysis catheters. #If a patient had multiple cardiothoracic surgeries, the time for the longest operative time was included.

No patient characteristics were significantly associated with increased odds of case status (Table 4). Odds of invasive NTM infection increased for progressively longer surgery times with HCD and longer time on bypass, although this reached statistical significance only for surgery time with HCD >5 hours and time on bypass >2 hours. Using the final logistic regression model in which surgery time with HCD was a dichotomous variable (models 1.1 and 1.2), the odds of NTM infection for surgical time with HCD >5 hours was 13.2 times and 13.6 times higher than for surgical time with HCD <5 hours, respectively, both without and with adjustment for immunocompromised status. Similarly, time on bypass for >2 hours (models 2.1 and 2.2) was associated with significantly higher odds of NTM infection both without (OR 16.5, 95% CI 3.8–84.0; p = 0.0004) and with (OR 16.6, 95% CI 3.8–88.4; p = 0.0006) adjustment for immunocompromised status.

**Table 4:** Results from logistic regression models to evaluate exposure length variables in investigation of invasive extrapulmonary NTM infections among patients who underwent cardiothoracic surgery, York, Pennsylvania, USA, 2015*

Regression models	Odds ratio (95% CI)	p value
Univariable models†		
Patient characteristics		
Age, per 1-y increase	1.0 (1.0–1.1)	0.4
Male sex	3.0 (0.7–16.8)	0.2
White race	1.8 (0.2–244.3)	0.7
Chronic lung disease: yes	2.9 (0.7–11.4)	0.1
Diabetes: yes	0.3 (0.03–1.5)	0.2
Immunocompromised: yes	1.5 (0.3–6.0)	0.6
Length of surgical exposure		
Surgical time with HCD, h		
0	Referent	
>0 to <4	1.8 (0.2–15.4)	0.6
≥4 to <5	2.6 (0.2–23.6)	0.4
≥5	15.6 (3.2–103.9)	0.002
Time on bypass, h		
0	Referent	
>0 to <2	1.7 (0.2–12.2)	0.6
≥2	19.0 (3.7–133.0)	0.001
Final logistic model‡		
Model 1.1, only surgical time with HCD retained in the final model: Surgical time with HCD >5 h	13.2 (3.2–62.9)	0.0008
Model 1.2, includes surgical time with HCD and immunocompromised status		
Surgical time with HCD >5h	13.6 (3.3–68.8)	0.001
Immunocompromised: yes	2.2 (0.4–12.2)	0.4
Model 2.1, only time on bypass retained in the final model: Time on bypass >2 h	16.5 (3.8–84.0)	0.0004
Model 2.2, includes time on bypass and immunocompromised status		
Time on bypass >2h	16.6 (3.8–88.4)	0.0006
Immunocompromised: yes	2.1 (0.3–12.0)	0.4

*HCD, heater–cooler device; NTM, nontuberculous mycobacteria. †Regression models for factors considered in the multivariable model; each regression model used Firth’s penalized maximum likelihood method. ‡Each final regression model used Firth’s penalized maximum likelihood method; other variables (sex, age, immunocompromised status, chronic lung disease) were removed because of nonsignificance.

### Infection Control and Environmental Assessment

**Figure 2:**
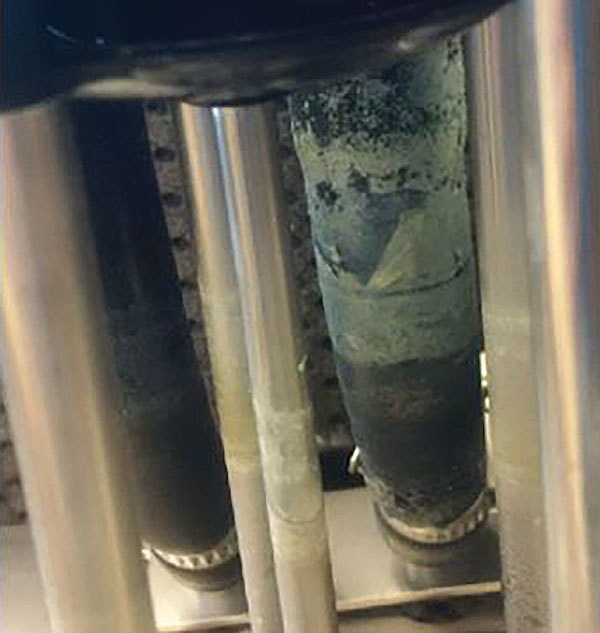
Biofilm visualized on surfaces submerged in an internal water reservoir of a heater–cooler device during investigation of invasive extrapulmonary nontuberculous mycobacteria infections among patients who underwent cardiothoracic surgery, York, Pennsylvania, USA, 2015.

An infection control assessment focusing on perioperative practices did not identify any breaches related to operating room ventilation, water use, storage, operating practices, and patient care. The hospital had 3 HCDs, all LivaNova (formerly Sorin Group Deutschland GmBH, Munich, Germany) Stӧckert Heater-Cooler 3T Systems (referred to as 3T HCDs); 1 was acquired in 2009 and 2 were acquired in 2012. The manufacturer revised its instructions for use in February 2015 and made additional revisions in a June 2015 field safety notice, including recommendations for more frequent and higher potency disinfection and for positioning the HCD so that its exhaust vent is directed away from the surgical field. Periodic updates to the manufacturer’s disinfection recommendations may have resulted in inconsistencies between the hospital’s HCD cleaning and disinfection practices before June 2015 and the manufacturer’s recommendations at that time. Before the onsite investigation, sterilized water had been used in HCD at the hospital, but all 3 HCDs were replaced and appropriate changes made to ensure compliance with the most recent manufacturer’s operating instructions. When a decommissioned HCD was dissembled for further inspection, biofilm was visible on tubing and surfaces submerged in an internal water reservoir (Figure 2).

### Laboratory Assessment

Water samples from all 3 decommissioned HCDs and swab specimens of the biofilm from the 1 disassembled HCD tested positive for *M. chimaera* (Table 5). Water samples from a scrub sink near the cardiothoracic operating rooms and ice machines used for nonsterile purposes in the operating room tested positive for rapid-growing NTM species but not *M. chimaera*. Culture results from water samples collected from the new HCD before installation were also negative. However, the concentrations of non-NTM bacteria detected in HCD water samples were higher after operating the device than before (150,000 vs. 116 colony-forming units/mL).

**Table 5:** Microbiologic test results for case-patient isolates and environmental samples in investigation of invasive extrapulmonary NTM infections among patients who underwent cardiothoracic surgery, York, Pennsylvania, USA, 2015*

Description	Sample type	Species identified
Patient isolates		
Case-patient 3	Blood	*M. chimaera*
Case-patient 4	Blood	*M. chimaera*
Case-patient 9	Blood	*M. chimaera*
Environmental samples		
HCD 1	Water	*M. chimaera*
HCD 2	Water/swab specimen	*M. chimaera*
HCD 3	Water	*M. chimaera*
Ice machine	Ice	*M. mucogenicum/M. phocaicum; M. gastri*
Scrub sink	Water	*M. avium*
Air sampling of HCD exhaust	Air	*M. chimaera*

*HCD, heater–cooler device; NTM, nontuberculous mycobacteria.

Air samples collected 18 inches from the HCD exhaust vent during the operating room simulations were found to be positive for *M. chimaera* after 2, 3, and 4 hours of HCD operation (Table 5). All remaining air samples, including those collected before starting the HCD, were negative for all NTM.

**Figure 3:**
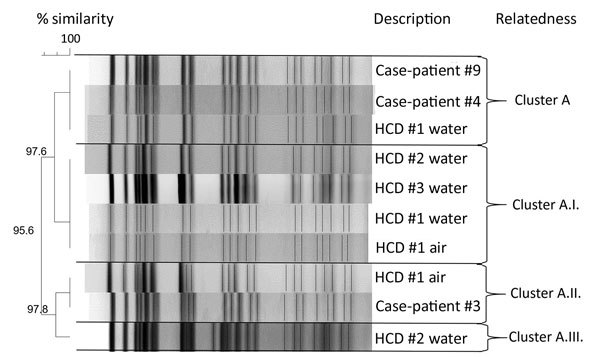
Molecular strain typing by pulsed-field gel electrophoresis of case-patients and environmental isolates of *Mycobacterium chimaera* in investigation of invasive extrapulmonary nontuberculous mycobacteria infections among patients who underwent cardiothoracic surgery, York, Pennsylvania, USA, 2015*.* HCD, heater–cooler device.

All 3 available case-patient isolates were identified as *M. chimaera*; these and the environmental *M. chimaera* isolates (obtained from air and HCD samples) were found to be highly related by PFGE (Table 5; Figure 3). Subsequent whole-genome sequencing results confirmed the PFGE analysis; *M. chimaera* sequences from clinical isolates, the HCDs, and air samples were highly related (*19*).

## Discussion

This investigation confirmed a prolonged outbreak of invasive MAC infections associated with cardiac surgery requiring cardiopulmonary bypass with exposure to 3T HCDs, similar to reports from Europe (*16*). The infection rate was low (8 cases/2,276 surgeries with HCDs) among those who were exposed, making recognition of this outbreak difficult. We describe a case–control study in which 8 case-patients likely had HCD-related *M. chimaera* and MAC infections. Laboratory testing suggested a common source of *M. chimaera* transmission from the HCDs through aerosolization, which is consistent with studies demonstrating NTM’s high propensity for aerosolization (*20*).

Our investigation had several limitations. Presentation with nonspecific symptoms and the low clinical suspicion by providers make diagnosis of invasive NTM infections difficult in this patient population. Challenges in diagnosis and clinical follow-up may have resulted in decreased sensitivity of case detection and misclassification bias. Misclassification of cases as controls is possible; patients may have received follow-up care at a different healthcare facility from the facility where surgery was performed. Because of the retrospective nature of this study and the prolonged period of the outbreak, it was not practical to obtain all potential health records from any healthcare facility at which patients may have been treated. Many clinical isolates from case-patients were unavailable, preventing further species identification of MAC specimens as *M. chimaera.* We used a maximum exposure window of ≈3.5 years for our analysis based on published reports available during the time of the investigation (*16*), but subsequent reports have suggested that the time between exposure and diagnosis can be as long as 6 years (*21*). However, preliminary review did not identify any additional patients with exposures >3.5 years before specimen collection who would have qualified for inclusion in this analysis. Our statistical analysis was limited by the small sample size and high correlation between various surgical exposures. Laboratory isolation of certain NTMs is hindered by NTM’s slow growth. Additionally, many clinical laboratories are unable to perform species-level identification of MAC to *M. chimaera* and would not be able to identify a cluster of infections caused by this organism.

Based on this investigation, CDC and the PADOH made several recommendations to the hospital to enhance detection and surveillance of HCD-related NTM infections and to mitigate risk during future cardiac surgeries. These recommendations included increasing awareness among patients and providers to facilitate earlier diagnosis and treatment. Wellspan York Hospital notified 1,300 cardiac surgery patients and their providers of the potential exposure and established an NTM clinic to evaluate and monitor these patients. Subsequently, an additional 4 patients with likely HCD-associated infections were identified. CDC and the PADOH also issued recommendations to mitigate the risk for HCD-related NTM infections prospectively by ensuring compliance with the manufacturer’s cleaning and disinfection instructions, positioning the HCD to minimize aerosolized particles from reaching patients, and using filter-sterilized water to decrease HCD contamination.

PADOH has been proactive in raising awareness of MAC infections related to the 3T HCDs, publicly reporting and investigating these initial cases and raising awareness of the issue among healthcare facilities and clinicians in Pennsylvania by issuing a statewide health advisory (*22*). Since October 2015, the Food and Drug Administration (FDA) and CDC have issued multiple nationwide communications to raise awareness, improve identification of contaminated HCDs and HCD-related infections, encourage notification of potentially exposed patients, and mitigate risk (*23*–*29*), including broad outreach to professional societies of providers caring for this patient population. CDC and FDA have continued to receive reports of NTM-contaminated devices and related infections with *M. chimaera*, and an FDA advisory panel was convened (*30*). Recent evidence suggests that contamination likely occurred during the manufacturing of the 3T HCDs; nearly identical strains of *M.* *chimaera* have been detected among samples from infected patients, HCDs from 3 different countries, and the 3T manufacturing plant (*31*). Whole-genome sequencing analysis of clinical and 3T HCD *M. chimaera* isolates from geographically distinct areas of the United States have demonstrated closely related strains, also suggesting point-source contamination of the 3T HCDs (*19*).

Additional areas for research include the effectiveness of disinfection practices given NTM’s propensity to form biofilm, as well as device design issues to decrease NTM growth and aerosolization (*30*). Hospitals and public health services should continue to raise provider and patient awareness about the risks of the 3T HCD (*25*). Short-term solutions to minimize risk, such as passing device exhaust through a HEPA filter or moving the device outside the operating room, may affect the design and functionality of the HCD and require careful examination (*28*). In addition, because clusters of extrapulmonary NTM infections may be a frequently underrecognized sentinel of medical device or environmental contamination that decreases the safety of surgical procedures, reporting of such infections to public health is a key patient safety measure.

In conclusion, our investigation confirmed an outbreak of MAC infections in which undergoing cardiac surgery requiring cardiopulmonary bypass with an HCD was associated with increased odds of infection, even in immunocompetent patients. Environmental sampling results suggest that airborne transmission occurred through aerosolization and dispersal of MAC while an HCD was operational. These findings highlight the need for increasing awareness of invasive NTM infection risk among cardiac surgery patients exposed to 3T HCDs; identifying best practices for notifying, evaluating, and managing potentially infected patients; and identifying options for mitigating infection risk from these devices.

**Table:** 

**Table 1.** Demographic and clinical details of case-patients with invasive extrapulmonary NTM infections following procedures using an HCD, York, Pennsylvania, USA, 2015*									
Patient no.	Age, y	Infection latency, y†	Year of first NTM specimen collection	Infection type	Location of NTM specimens	NTM organism	Bypass (year of surgery)	Time on bypass, h	Death‡
1	80–90	2.3	2010	Thoracic	Pleural fluid	*M. kansasii*	Yes (2008)	2–3	Yes
2	70–80	0.1	2010	Thoracic	Pleural fluid	MAC	Yes (2010)	3–4	Yes
3	40–50	1.7	2011	Extrathoracic	Blood, tissue from port site	*M. mucogenicum/* *M. fortiutum*	No	NA	Yes
4	60–70	0.7	2012	Extrathoracic	Bone marrow, spleen	*M. chimaera*	Yes (2012)	2–3	Yes
5	70–80	2.0	2012	Extrathoracic	Liver, bone marrow	*M. chimaera*	Yes (2010)	2–3	No
6	70–80	0.5	2012	Extrathoracic	Blood, bone marrow	MAC	Yes (2012)	2–3	Yes
7	60–70	0.8	2014	Thoracic	Deep sternal wound§	*Mycobacterium* species	No	NA	No
8	60–70	1.9	2014	Thoracic	Deep sternal wound§	MAC	Yes (2012)	<1	No
9	80–90	1.4	2014	Extrathoracic	Psoas abscess¶	MAC	Yes (2013)	1–2	No
10	60–70	0.5	2015	Extrathoracic	Bone marrow	*M. chimaera*	Yes (2014)	2–3	Yes
11	60–70	1.4	2015	Thoracic	Pleural fluid	MAC	Yes (2013)	2–3	Yes

*HCD, heater–cooler device; MAC, *Mycobacterium avium* complex; NA, not available; NTM, nontuberculous mycobacteria. †Length of time in years between first NTM-positive specimen and most invasive cardiothoracic surgery ‡Death was not necessarily attributable to NTM infection. §Communicating with pleural space. ¶With radiographic findings of adjacent vertebral osteomyelitis.
